# The Influence of Solvent on the Crystal Packing of Ethacridinium Phthalate Solvates

**DOI:** 10.3390/ma13225073

**Published:** 2020-11-10

**Authors:** Artur Mirocki, Artur Sikorski

**Affiliations:** Faculty of Chemistry, University of Gdańsk, W. Stwosza 63, 80-308 Gdańsk, Poland

**Keywords:** ethacridine, phthalic acid, hydrogen bonds, π–π stacking interactions, crystal packing, supramolecular synthons

## Abstract

The synthesis, structural characterization and influence of solvents on the crystal packing of solvated complexes of ethacridine with phthalic acid: 6,9-diamino-2-ethoxyacridinium phthalate methanol solvate (**1**), 6,9-diamino-2-ethoxyacridinium phthalate ethanol solvate (**2**), 6,9-diamino-2-ethoxyacridinium phthalate isobutanol solvate (**3**), and 6,9-diamino-2-ethoxyacridinium phthalate *tert*-butanol solvate monohydrate (**4**) are described in this article. Single-crystal XRD measurements revealed that the compounds **1**–**4** crystallized in the triclinic *P*-1 space group, and the 6,9-diamino-2-ethoxyacridinium cations, phthalic acid anions and solvent molecules interact via strong N–H···O, O–H···O, C–H···O hydrogen bonds, and C–H···π and π–π interactions to form different types of basic structural motifs, such as: heterotetramer *bis*[···cation···anion···] in compound **1** and **2**, heterohexamer *bis*[···cation···alcohol···anion···] in compound **3**, and heterohexamer *bis*[···cation···water···anion···] in compound **4**. Presence of solvents molecule(s) in the crystals causes different supramolecular synthons to be obtained and thus has an influence on the crystal packing of the compounds analyzed.

## 1. Introduction

6,9-Diamino-2-ethoxyacridine (common name: ethacridine) is an active pharmaceutical ingredient (API) having a broad range of activity due to the ability to intercalate to DNA [1]. A commonly available drug, ethacridine lactate monohydrate (acrinol) exhibits antiviral properties and is helpful in curing suppurating infections, inflamed wounds, burns, as well as local infections of the mouth and throat, and inhibits protein synthesis in bacterial cells [2,3]. Acrinol causes the death of thyroid cancer cells [4], also, it finds a wide spectrum of other applications [5,6,7].

From a structural point of view, ethacridine is a poorly known compound. A search of the Cambridge Structure Database (CSD version 5.41, update March 2020) shows that there are only six known crystal structures containing the ethacridinium cation, including ethacridinium lactate monohydrate (REFCODE: BIMJUC) [8], two polymorphs of ethacridinium lactate (REFCODE: COVSUD, COVZOE) [9] and three dihydrates of ethacridinium halobenzoates: 3-chlorobenzoate, 3-bromobenzoate, and 3-iodobenzoate [10]. The reason for such a small number of structures is the difficulty of obtaining single crystals of high purity and appropriate quality of XRD experiments.

Our previous research on crystals containing acridine derivatives [10,11,12,13] shows that benzoic acids are good candidates for the preparation of multi-component crystals containing these APIs. Moreover, it is known that the use of different solvents during the crystallization process provide the yield of a different solvates of multi-component crystals involving APIs [14,15,16,17,18,19,20,21,22,23], including acridine derivatives [24]. Taking into account the structures of crystals, there are two reasons for the formation of API solvates [25,26]. The main driving force is occurrence of the various intermolecular interactions between solvent molecule(s) and other components in these crystals, including hydrogen bonds, e.g., N–H···O [27,28,29], O–H···O [30,31], C–H···O [32,33], and other interactions, e.g., C–H···π [34,35,36,37], π–π [38,39], lp···π [40,41], which influences the self-assembly processes of APIs. It is also known that the presence of solvent molecules decreases the void space in the crystal lattice [25,26].

Considering the above, in this paper, we describe the synthesis and structural characterization of four solvated complexes of ethacridine (6,9-diamino-2-ethoxyacridine) with phthalic acid, prepared using different solvents (methanol, ethanol, isobutanol, *tert*-butanol). In addition, the analysis of intermolecular interactions and discussion on the solvent influence on the crystal packing of title compound are presented.

## 2. Materials and Methods

All the chemicals were purchased from Sigma-Aldrich (St. Louis, MO, USA) and used without further purification. Melting points were determined on a Buchi 565 capillary apparatus and were uncorrected.

### 2.1. Synthesis of Compounds **1**–**4**

(1) 6,9-Diamino-2-ethoxyacridinium phthalate methanol solvate (6,9-diamino-2-ethoxyacridinium phthalate–methanol (1/1)) (**1**)

Ethoxyacridine-DL-lactate monohydrate (0.05 g, 0.138 mmol) and phthalic acid (0.046 g, 0.277 mmol) were dissolved in 15 cm^3^ of a methanol and boiled for ca. 20 min. After cooling, a few drops of dichloromethane were added to the mixture. The solution was allowed to evaporate for a few days to give yellow crystals of **1** (yield ca. 90%, m.p. = 237.1 °C).

(2) 6,9-Diamino-2-ethoxyacridinium phthalate ethanol solvate (6,9-diamino-2-ethoxyacridinium phthalate–ethanol (1/1)) (**2**).

Ethoxyacridine-DL-lactate monohydrate (0.05 g, 0.138 mmol) and phthalic acid (0.023 g, 0.138 mmol) were dissolved in 15 cm^3^ of an ethanol/water mixture (2:1 *v/v*) and boiled for ca. 20 min. The solution was allowed to evaporate for a few days to give yellow crystals of **2** (yield ca. 90%, m.p. = 237.4 °C).

(3) 6,9-Diamino-2-ethoxyacridinium phthalate isobutanol solvate (6,9-diamino-2-ethoxyacridinium phthalate–isobutanol (1/1)) (**3**)

Ethoxyacridine-DL-lactate monohydrate (0.05 g, 0.138 mmol) and phthalic acid (0.023 g, 0.138 mmol) were dissolved in 15 cm^3^ of an isobutanol/water mixture (2:1 *v/v*) and boiled for ca. 20 min. The solution was allowed to evaporate for a few days to give yellow crystals of **3** (yield ca. 90%, m.p. = 187.1 °C)

(4) 6,9-Diamino-2-ethoxyacridinium phthalate *tert*-butanol solvate monohydrate (6,9-diamino-2-ethoxyacridinium phthalate–*tert*-butanol–water (1/1/1)) (**4**)

Ethoxyacridine-DL-lactate monohydrate (0.05 g, 0.138 mmol) and phthalic acid (0.023 g, 0.138 mmol) were dissolved in 15 cm^3^ of a *tert*-butanol/water mixture (2:1 *v/v*) and boiled for ca. 20 min. The solution was allowed to evaporate for a few days to give yellow crystals of **4** (yield ca. 70%, m.p. = 140.3 °C).

### 2.2. X-ray Measurements and Refinements

Diffraction data were collected on an Oxford Diffraction Gemini R ULTRA Ruby CCD diffractometer (T = 295(2) K, MoK_α_ (λ = 0.71073 Å) radiation, Table 1) and were reduced using CrysAlis RED software (ver. 1.171.41.16a) [42]. The structures were refined and solved using the SHELX package (ver. 2017/1) [43]. The solvent molecules in compounds **1**–**4**, i.e., methanol, ethanol, isobutanol and *tert*-butanol molecules respectively, have orientation disorders (refined site-occupancy factors of the disordered parts are: 0.78(3) and 0.22(3) for compound **1**, 0.73(1) and 0.27(1) for compound **2**, 0.62(1) and 0.38(1) for compound **3** and 0.77(1) and 0.23(1) for compound **4**). H-atoms bound to nitrogen or oxygen atoms were located on a difference Fourier map and refined freely, whereas other H-atoms were placed geometrically with *d*_(C–H)_ = 0.93–0.98 Å and U_iso_(H) = 1.2–1.5U_eq_(C). All interactions were identified using the PLATON program (ver. 181115) [44], while the ORTEPII [45], PLUTO-78 [46] and Mercury (ver. 2020.2.0) [47] programs were used to prepare the molecular graphics. For clarity, disordered parts of the solvents were omitted from the illustrations of the molecular structure and crystal packing.

## 3. Results

### 3.1. Crystal Structure of Ethacridinium Phthalate Methanol Solvate (**1**)

**Figure 1:**
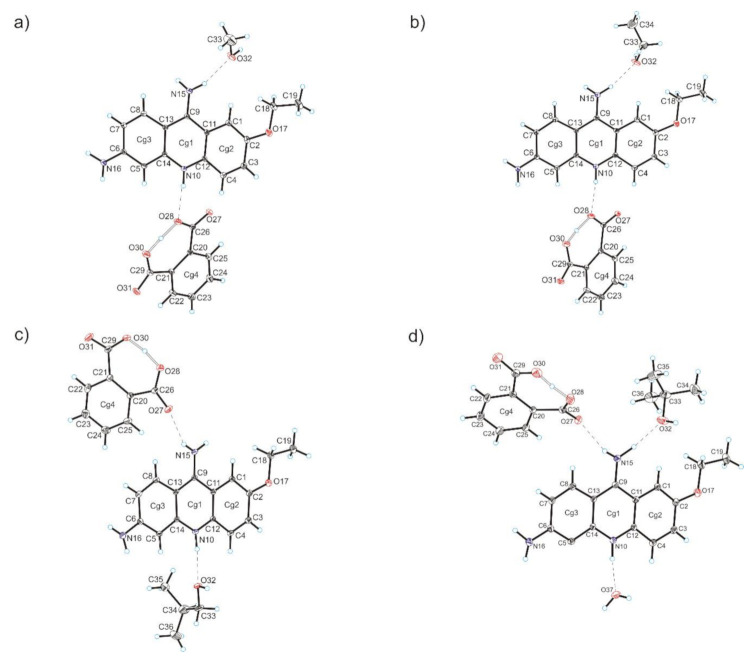
Crystal structures of compounds **1**–**4** (**a**–**d**) with the atom-labelling scheme (hydrogen bonds are represented by dashed lines). Cg1, Cg2, Cg3 are the ring centroids.

**Figure 2:**
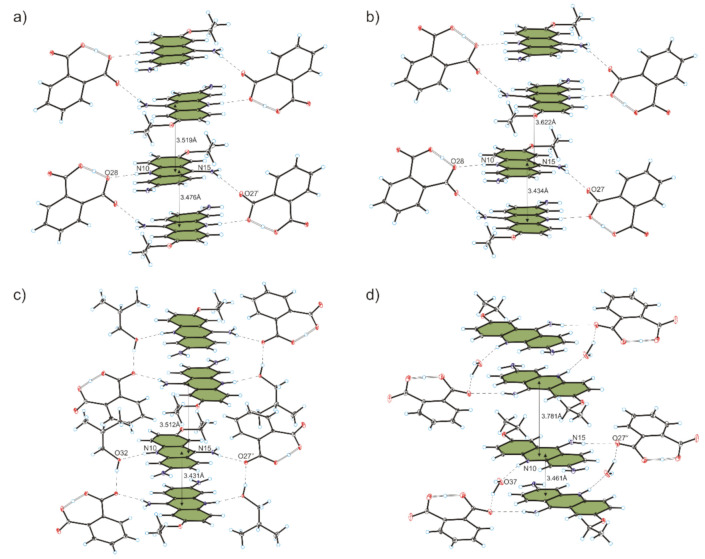
Heterotetramers in compounds **1**–**2** and heterohexamers in compounds **3**–**4** shown in (**a**,**b**), and (**c**,**d**), respectively.

**Figure 3:**
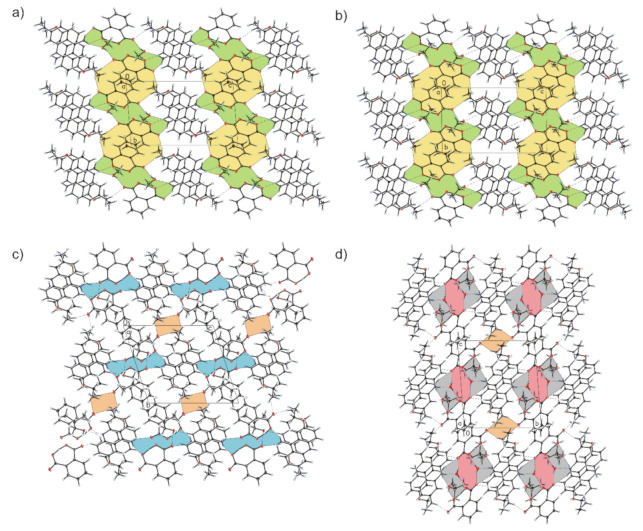
Crystal packing and supramolecular synthon of compounds **1**–**4** shown in (**a**–**d**) respectively. (**a**,**b**) Supramolecular synthons: [⋯H–N–H⋯(O
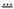
C
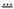
O
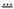
H
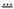
O
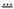
C
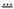
O)^−^⋯]_2_ (highlighted in yellow), and [⋯H–N–H⋯O–H⋯(O
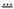
C
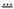
O
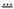
H
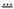
O
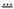
C
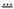
O)^−^⋯]_2_ (highlighted in green). (**c**) Supramolecular synthons: [⋯H–N–H⋯(O
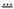
H
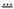
O
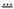
C
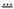
O)^−^⋯]_2_ (highlighted in blue), and [⋯H–N–H⋯O⋯]_2_ (highlighted in orange). (**d**) Supramolecular synthons: [⋯H–N–H⋯O⋯]_2_ (highlighted in orange), [⋯H–O–H⋯O
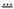
C
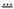
O⋯]_2_ (highlighted in pink), and [⋯H–N–H⋯O–H⋯(O
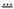
H
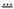
O
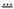
C
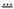
O)^−^⋯]_2_ (highlighted in gray).

Compound **1** crystallizes in the triclinic *P-*1 space group with 6,9-diamino-2-etoxyacridinium cation, phthalate anion and methanol molecule in the asymmetric unit (Figure 1a). The C—O carboxylic acid bond lengths (1.266(3) Å–1.281(3) Å) reveal that a proton transfer occurring between the carboxylic group of phthalic acid and the endocyclic N-atom of 6,9-diamino-2-etoxyacridine. In the monoprotonated phthalate anion, the H-atom is shared between two O-atoms from the two carboxylate groups, and we observe an intramolecular O_(carboxy)_–H···O_(carboxy)_ hydrogen bond [*d*(O28···O30) = 2.406(2) Å, *d*(H28···O30) = 1.15(3) Å]. The 6,9-diamino-2-etoxyacridine cation is linked with phthalic acid anion by N_(acridine)_–H···O_(carboxy)_ [*d*(H10···O28) = 1.96(2) Å and ∠(N10–H10···O28) = 172(3)°], and N_(9-amino)_–H···O_(carboxy)_ [*d*(H15A···O27) = 2.11(3) Å and ∠(N15–H15A···O27) = 155(2)°] hydrogen bonds to form a cyclic heterotetramer (Table 2, Figure 2a) [48,49]. Moreover, this heterotetramer is stabilized through N_(9-amino)_–H···O_(methanol)_ hydrogen bond between the 6,9-diamino-2-etoxyacridine cation and the methanol molecule [*d*(H15B···O32) = 2.05(3) Å and ∠(N15–H15B···O32) = 159(2)°]. We also observe the weak C_(acridine)_–H···O_(methanol)_ hydrogen bond involving C-1 atom of 6,9-diamino-2-etoxyacridine cation and an oxygen atom from hydroxyl group of methanol molecule [*d*(H1···O32A) = 2.56 Å and ∠(C1–H1···O32A) = 161°]. Adjacent 6,9-diamino-2-etoxyacridine cations engaged in the formation of heterotetramer interact through π–stacking interactions with distance between centroids [*d*(Cg···Cg)] ranging from 3.689(2) Å to 4.001(2) Å and separation between the mean planes of the 6,9-diamino-2-etoxyacridine skeleton from 3.476 Å to 3.519 Å (Table 3). Adjacent π–stacked heterotetramers interact via N_(6-amino)_–H···O_(carboxy)_ hydrogen bonds among amino group in position C-6 from the 6,9-diamino-2-etoxyacridine cation and carboxylic groups form phthalic acid anions [*d*(H16A···O27) = 2.05(3) Å and ∠(N16–H16A···O27) = 163(2)°, and *d*(H16B···O31) = 2.12(3) Å and ∠(N16–H16B···O31) = 167(2)°] and O_(methanol)_–H···O_(carboxy)_ hydrogen bond between methanol molecule and phthalic acid anion [*d*(H32···O31) = 1.90(6) Å and ∠(O32–H32···O31) = 160(6)°] (Figure 3a). Neighbouring 6,9-diamino-2-etoxyacridine cations are also connected by C_(acridine)_–H···π interactions [*d*(H19C···Cg3) = 3.16 Å and ∠(C19–H19C···Cg3) = 136°, and *d*(H18A···Cg1) = 3.43 Å and ∠(C18–H18A···Cg1) = 127°, and *d*(H18A···Cg3) = 3.41 Å and ∠(C18–H18A···Cg3) = 108°] to form a three-dimensional framework structure.

### 3.2. Crystal Structure of Ethacridinium Phthalate Ethanol Solvate (**2**)

Compound **2** crystallizes in the triclinic *P*-1 space group with 6,9-diamino-2-etoxyacridinium cation, phthalate anion and ethanol molecule in the asymmetric unit (Figure 1b). The C—O carboxylic acid bond lengths (1.270(2) Å–1.280(2) Å), reveal that a proton transfer occurring between the carboxylic acid group and the endocyclic N-atom of 6,9-diamino-2-etoxyacridine. In the monoprotonated phthalate anion, the H-atom is divided between two O-atoms from both carboxylate groups. There is an intramolecular O_(carboxy)_–H···O_(carboxy)_ hydrogen bond [*d*(O28···O30) = 2.392(2) Å and *d*(H28···O30) = 1.08(3) Å]. The 6,9-diamino-2-etoxyacridine cation is linked with the phthalic acid anion via N_(acridine)_–H···O_(carboxy)_ [*d*(H10···O28) = 2.00(2) Å and ∠(N10–H10···O28) = 171(2)°] and N_(9-amino)_–H···O_(carboxy)_ [*d*(H15A···O27) = 2.10(2) Å and ∠(N15–H15A···O27) = 154(2)°] hydrogen bonds to form a cyclic heterotetramer, similar to the previous example (Table 2, Figure 2b). Adjacent heterotetramers feature π–stacking formed between 6,9-diamino-2-etoxyacridine cations [*d*(Cg···Cg) = 3.615(1) Å–3.800(1) Å and separation from 3.434 Å to 3.622 Å (Table 3)]. The N_(9-amino)_–H···O_(ethanol)_ hydrogen bond between the 6,9-diamino-2-etoxyacridine cation and the ethanol molecule [*d*(H15B···O32) = 2.03(2) Å and ∠(N15-H15B···O32) = 163(2)°], and weak C_(acridine)_–H···O_(ethanol)_ hydrogen bond involving the C-1 atom of 6,9-diamino-2-etoxyacridine cation and an the O-atom from the hydroxyl group of ethanol molecule [*d*(H1···O32) = 2.58 Å and ∠(C1–H1···O32) = 157°] stabilize these heterotetramers. The neighbouring π–stacked heterotetramers interact through N_(6-amino)_–H···O_(carboxy)_ hydrogen bonds between amino group in position C-6 from the 6,9-diamino-2-etoxyacridine cation and carboxylic groups of phthalate anion [*d*(H16B···O27) = 2.14(3) Å and ∠(N16–H16B···O27) = 164(2)°, and *d*(H16A···O31) = 2.09(2) Å and ∠(N16–H16A···O31) = 168(2)°], and O_(ethanol)_–H···O_(carboxy)_ hydrogen bond between the ethanol molecule and the phthalic acid anion [*d*(H32···O31) = 2.23 Å and ∠(O32–H32···O31) = 126°] (Figure 3b). The 6,9-diamino-2-etoxyacridine cations feature C_(acridine)_–H···π interactions between each other [*d*(H18A···Cg1) = 3.07 Å and ∠(C18–H18A···Cg1) = 145°, and *d*(H19C···Cg3) = 2.94 Å and ∠(C19–H19C···Cg3) = 135°] forming a 3D framework structure.

### 3.3. Crystal Structure of Ethacridinium Phthalate Isobutanol Solvate (**3**)

Compound **3** crystallizes in the triclinic *P*-1 space group with 6,9-diamino-2-etoxyacridinium cation, phthalate anion and isobutanol molecule in the asymmetric unit (Figure 1c). The C—O carboxylic acid bond lengths (1.271(2) Å–1.284(3) Å), reveal that a proton transfer occurring between the carboxylic acid group and the endocyclic N-atom of 6,9-diamino-2-etoxyacridine. In the monoprotonated phthalate anion, the H-atom is shared between two O-atoms from the two carboxylate groups and intramolecular O_(carboxy)_–H···O_(carboxy)_ hydrogen bond [*d*(O28···O30) = 2.388(2) Å and *d*(H28···O30) = 1.19(3) Å] is observed. In the crystals of compound **3**, the 6,9-diamino-2-etoxyacridine cation interacts with the phthalic acid anion via N_(9-amino)_–H···O_(carboxy)_ [*d*(H15A···O27) = 2.13(2) Å and ∠(N15–H15A···O27) = 150(2)°] hydrogen bond, whereas one isobutanol molecule interacts with both the 6,9-diamino-2-etoxyacridine cation and the phthalic acid anion through N_(acridine)_–H···O_(isobutanol)_ [*d*(H10···O32) = 1.96(2) Å and ∠(N10–H10···O32) = 169(2)°], and O_(isobutanol)_–H···O_(carboxy)_ [*d*(H32···O27) = 1.94 Å and ∠(O32–H32···O27) = 162°] hydrogen bonds to form a centrosymmetric heterohexamer (Table 2, Figure 2c) [50]. Neighbouring 6,9-diamino-2-etoxyacridine cations involved in the formation of heterohexamer interact via π–stacking interactions [*d*(Cg···Cg) = 3.568(1) Å–3.948(1) Å and separation from 3.431 Å to 3.512 Å (Table 3)]. Adjacent π–stacked heterohexamers are linked via N_(9-amino)_–H···O_(carboxy)_ hydrogen bond between the 6,9-diamino-2-etoxyacridine cation and the phthalate anion [*d*(H15B···O30) = 2.04(3) Å and ∠(N15–H15B···O30) = 153(2)°] and N_(6-amino)_–H···O_(carboxy)_ hydrogen bonds involving the amino group in position C-6 from the 6,9-diamino-2-etoxyacridine cation and carboxylic groups of the phthalic acid anion [*d*(H16A···O31) = 2.06(2) Å and ∠(N16–H16A···O31) = 168(2)°, and *d*(H16B···O31) = 2.53(3) Å and ∠(N16–H16B···O31) = 123(2)°] (Figure 3c). Adjacent 6,9-diamino-2-etoxyacridine cations are also connected by C_(acridine)_–H···π interactions [*d*(H18B···Cg1) = 2.85 Å and ∠(C18–H18B···Cg1) = 135°, and *d*(H19B···Cg3) = 2.86 Å and ∠(C19–H19B···Cg3) = 147°] to form a three-dimensional framework structure.

### 3.4. Crystal Structure of Ethacridinium Phthalate Tert-Butanol Solvate Monohydrate (**4**)

Compound **4** crystallizes in the triclinic *P*-1 space group with 6,9-diamino-2-etoxyacridinium cation, phthalate anion, *tert*-butanol molecule, and water molecule in the asymmetric unit (Figure 1d). The C—O carboxylic acid bond lengths (1.274(4) Å–1.289(4) Å), reveal that a proton transfer occurring between the carboxylic acid group and the endocyclic N-atom of 6,9-diamino-2-etoxyacridine. In the monoprotonated phthalate anion, the H-atom is divided between two O-atoms from the two carboxylate groups and we observe an intramolecular O_(carboxy)_–H···O_(carboxy)_ hydrogen bond [*d*(O28···O30) = 2.371(6) Å and *d*(H28···O30) = 1.11(6) Å]. Cation of 6,9-diamino-2-etoxyacridine interact with the phthalic acid anion via N_(9-amino)_–H···O_(carboxy)_ hydrogen bond [*d*(H15A···O27) = 2.33(4) Å and ∠(N15–H15A···O27) = 162(3)°], while water molecule interacts with both the 6,9-diamino-2-etoxyacridine cation and the phthalic acid anion by N_(acridine)_–H···O_(carboxy)_ [*d*(H10···O37) = 1.87(4) Å and ∠(N10–H10···O37) = 164(3)°], and O_(water)_–H···O_(carboxy)_ [*d*(H37A···O27) = 2.18(7) Å and ∠(O37–H37A···O27) = 165(8)°] hydrogen bonds, to form a centrosymmetric heterohexamer (Table 2, Figure 2d). Adjacent 6,9-diamino-2-etoxyacridine cations interact by π–stacking [*d*(Cg···Cg) = 3.618(2) Å–3.743(2) Å and separation from 3.461 Å to 3.781 Å (Table 3)]. The neighbouring π–stacked heterohexamers are linked by O_(water)_–H···O_(carboxy)_ hydrogen bond between water molecules and phthalate anion [*d*(H37B···O28) = 1.97(7) Å and ∠(O37–H37B···O28) = 163(6)°], by the N_(9-amino)_–H···O_(*t*-butanol)_ hydrogen bond between the 6,9-diamino-2-etoxyacridine cations and the *tert*-butanol molecules [*d*(H15B···O32) = 1.97(4) Å and ∠(N15–H15B···O32) = 165(3)°], and through O_(*t*-butanol)_–H···O_(carboxy)_ hydrogen bond [*d*(H32···O30) = 2.57 Å and ∠(O32–H32···O30) = 154°] between the *tert*-butanol molecules and the phthalic acid anion. The neighbouring heterohexamers are also directly connected by N_(6-amino)_–H···O_(carboxy)_ hydrogen bonds involving the amino group in position C-6 from the 6,9-diamino-2-etoxyacridine cation and carboxylic groups of the phthalic acid anions [*d*(H16A···O31) = 2.51(5) Å and ∠(N16–H16A···O31) = 150(3)°, and *d*(H16B···O31) = 2.09(5) Å and ∠(N16–H16B···O31) = 169(4)°]. Weak C_(acridine)_–H···O_(carboxy)_ hydrogen bond between the 6,9-diamino-2-etoxyacridine cation and the phthalic anion is also observed [*d*(H8···O27) = 2.45 Å, and ∠(C8–H8···O27) = 174°] (Figure 3d). We also observed that neighbouring 6,9-diamino-2-etoxyacridine cations interact with each other by C_(acridine)_–H···π interactions [*d*(H18B···Cg3) = 2.87 Å and ∠(C18–H18C···Cg3) = 143°, and *d*(H19B···Cg3) = 3.65 Å and ∠(C19–H19C···Cg3) = 98°] forming a 3D framework structure.

## 4. Discussion

Comparing crystal data for compounds (Table 1), revealed that compounds **1**–**4** crystallized in the triclinic *P*-1 space group. However, only 6,9-diamino-2-ethoxyacridinium phthalate methanol solvate (**1**) and 6,9-diamino-2-ethoxyacridinium phthalate ethanol solvate (**2**) are isostructural, and none of the structures **1**–**4** are isostructural with ethacridinium: lactate (triclinic *P*-1 or monoclinic *C*2/c space groups), lactate monohydrate (triclinic *P*-1 space group) [8,9], or *meta*-halobenzoates dihydrates (monoclinic *P*2_1_/c space group) [10]. In the crystals of compounds **1** and **2** the basic structural motif is the cyclic heterotetramer *bis*[···cation···anion···] (Figure 2a,b and Table 2). This heterotetramer is created as a result of N_(9-amino)_–H···O_(carboxy)_ and N_(acridine)_–H···O_(carboxy)_ hydrogen bonds between 6,9-diamino-2-etoxyacridine cations and phthalic acid anions, and none of the heterotetramers contains any alcohol molecules. This heterotetramer is stabilized via π–stacking (Table 3); however, the distance between the mean plane of the acridine skeleton is smaller for compound **2**, and the distance between the mean plane of acridine skeleton of adjacent heterotetramers is smaller for compound **1**. A different situation arises in the case of crystals of 6,9-diamino-2 ethoxyacridinium phthalate isobutanol solvate (**3**) and 6,9-diamino-2-ethoxyacridinium phthalate *tert*-butanol solvate monohydrate (**4**) (Figure 2c,d and Table 2). Here, the basic structural motif is the cyclic heterohexamer, previously observed in the crystals of 6,9-diamino-2-etoxyacridium *meta*-halobenzoates dihydrates [10], yet they differ from each other. In the crystal of compound **3**, the 6,9-diamino-2-etoxyacridine cation interacts with the phthalic acid anion through the N_(9-amino)_–H···O_(carboxy)_ hydrogen bond, whereas one isobutanol molecule interacts with both 6,9-diamino-2-etoxyacridine cation and the phthalic acid anion via N_(acridine)_–H···O_(isobutanol)_ and O_(isobutanol)_–H···O_(carboxy)_ hydrogen bonds, to produce a centrosymmetric heterohexamer *bis*[···cation···isobutanol···anion···]. In the crystal of compound **4**, the 6,9-diamino-2-etoxyacridine cation interacts with the phthalic acid anion through the N_(9-amino)_–H···O_(carboxy)_ hydrogen bond, whereas one water molecule interacts with both the 6,9-diamino-2-etoxyacridine cation and the phthalic acid anion via N_(acridine)_–H···O_(water)_ and O_(water)_–H···O_(carboxy)_ hydrogen bonds respectively, to form a centrosymmetric heterohexamer *bis*[···cation···water···anion···]. In crystals **3** and **4**, the neighbouring heterohexamers are connected by π–stacking interactions between aromatic rings of acridine moieties (Table 3). The distances between the mean plane of the acridine skeleton and of neighbouring heterotetramers are smaller for compound **3** than for compound **4** in these heterohexamers. The crystal of **3** is the only one that contains an alcohol molecule, whereas the crystal of compound **4** is the only one that contains a water molecule in its basic structural motifs (heterotetramers or heterohexamers).

Although all of the compounds **1**–**4** crystallize in the triclinic *P*-1 space group, and the adjacent heterotetramers (**1** and **2**), or heterohexamers (**3** and **4**) form stacks (Figure 3), we can observe different supramolecular synthons in the crystal packing of compounds analysed. In the crystals of ethacridinium phthalate methanol solvate (**1**) and ethacridinium phthalate ethanol solvate (**2**), the neighbouring stacks are connected by N_(6-amino)–_H···O_(carboxy)_ hydrogen bonds involving the amino group in position C-6 from the 6,9-diamino-2-etoxyacridine cation and the carboxylic group form the phthalic acid anion and create supramolecular cyclic synthons [⋯H–N–H⋯(O
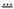
C
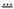
O
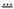
H
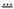
O
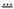
C
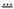
O)^−^⋯]_2_ (the 20-membered ring) [51,52,53] (Figure 3a,b). There are also other supramolecular cyclic synthons [⋯H–N–H⋯O–H⋯(O
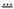
C
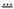
O
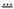
H
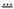
O
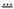
C
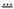
O)^−^⋯]_2_ (the 24-membered ring) which are created by N_(9-amino)–_H···O_(methanol)_ hydrogen bonds between the amino group in position C-9 from the 6,9-diamino-2-etoxyacridine cation and the methanol molecule, O_(methanol)–_H···O_(carboxy)_ hydrogen bonds between the methanol molecule and the carboxylic group form the phthalic acid anion, and N_(9-amino)–_H···O_(carboxy)_ hydrogen bonds including the amino group in position C-9 from the 6,9-diamino-2-etoxyacridine cation and oxygen atom of the carboxylate group (Figure 3a,b). In the crystal structure of ethacridinium phthalate isobutanol solvate (**3**) we observed supramolecular cyclic synthons [⋯H–N–H⋯(O
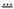
H
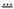
O
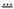
C
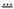
O)^−^⋯]_2_ (the 16-membered ring) (Figure 3c). This synthon is formed by N_(9-amino)–_H···O_(carboxy)_ hydrogen bonds between the amino group in position C-9 from the 6,9-diamino-2-etoxyacridine cation and the carboxylic groups form the phthalic acid anion. The neighbouring stacks are also connected by N_(6-amino)_–H···O_(carboxy)_ hydrogen bonds between the amino group in position C-6 from the 6,9-diamino-2-etoxyacridine cation and the carboxylic group form the phthalic acid anion hence creating supramolecular cyclic synthons [⋯H–N–H⋯O⋯]_2_ (the 8-membered ring) (Figure 3c). The same cyclic synthons appear in the crystal structure of 6,9-diamino-2-ethoxyacridinium phthalate *tert*-butanol solvate monohydrate (**4**) (Figure 3d). Furthermore, there are supramolecular cyclic synthons [⋯H–O–H⋯O
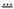
C
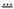
O⋯]_2_ (the 12-membered ring) created via O_(water)_–H···O_(carboxy)_ hydrogen bonds involving water molecules and the carboxylic group form the phthalic acid anion, which are inside another, bigger supramolecular cyclic synthons [⋯H–N–H⋯O–H⋯(O
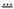
H
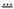
O
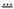
C
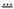
O)^−^⋯]_2_ (the 20-membered ring) (Figure 3d).

## 5. Conclusions

The synthesis, crystal structures and solvent influence on the crystal packing of ethacridinium phthalate solvates: 6,9-diamino-2-ethoxyacridinium phthalate methanol solvate (**1**), 6,9-diamino-2-ethoxyacridinium phthalate ethanol solvate (**2**), 6,9-diamino-2-ethoxyacridinium phthalate isobutanol solvate (**3**) and 6,9-diamino-2-ethoxyacridinium phthalate *tert*-butanol solvate monohydrate (**4**) are described in this article. Single-crystal XRD measurements revealed that the title compounds crystallized in the triclinic *P*-1 space group. However, only crystals of **1** and **2** are isostructural, while none of the structures **1**–**4** are isostructural with the known crystal structures of ethacridinium salts deposited in the CSD. The presence of solvents molecules in the crystals has an influence on the crystal packing of multicomponent crystals. In the crystal structure of compounds analyzed the 6,9-diamino-2-ethoxyacridinium cations, phthalic acid anions and solvent molecules interact through N–H···O, O–H···O, and C–H···O hydrogen bonds, as well as C–H···π, and π–π interaction, to form different types of basic structural motifs, such as: heterotetramer *bis*[···cation···anion···] in compound **1** and **2**, heterohexamer *bis*[···cation···alcohol···anion···] in compound **3**, and heterohexamer *bis*[···cation···water···anion···] in compound **4**. We also observed different supramolecular synthons depending on solvent molecule(s) in the crystal packing: [⋯H–N–H⋯(O
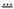
C
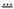
O
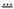
H
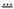
O
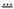
C
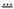
O)^−^⋯]_2_ (the 20-membered ring) and [⋯H–N–H⋯O–H⋯(O
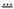
C
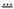
O
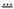
H
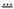
O
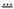
C
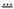
O)^−^⋯]_2_ (the 24-membered ring) in compounds **1** and **2**; [⋯H–N–H⋯(O
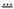
H
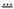
O
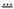
C
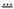
O)^−^⋯]_2_ (the 16-membered ring) in compound **3**; [⋯H–N–H⋯O⋯]_2_ (the 8-membered ring) in compounds **3** and **4**; [⋯H–O–H⋯O
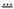
C
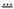
O⋯]_2_ (the 12-membered ring) and [⋯H–N–H⋯O–H⋯(O
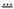
H
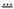
O
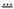
C
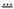
O)^−^⋯]_2_ (the 20-membered ring) in compound **4**. This research is a part of work aiming to determine the influence of different benzoic acid molecules, on the crystal packing of multicomponent crystals formed from ethacridine, with the use of different solvents.

## Figures and Tables

**Table 1:** Crystal data and structure refinement for compounds **1**–**4.**

Compound	1	2	3	4
Chemical formula	C_24_H_25_N_3_O_6_	C_25_H_27_N_3_O_6_	C_27_H_31_N_3_O_6_	C_27_H_32_N_3_O_7_
Formula weight/g·mol^−1^	451.47	465.49	493.55	510.56
Crystal system	triclinic	triclinic	triclinic	triclinic
Space group	*P*-1	*P*-1	*P*-1	*P*-1
*a*/Å	8.6152(11)	8.8199(7)	9.7818(6)	8.2878(7)
*b*/Å	9.1061(9)	9.2600(5)	11.5275(7)	12.2101(12)
*c*/Å	14.7634(14)	14.5715(9)	12.7367(7)	13.4549(16)
*α*/°	85.129(8)	88.489(5)	68.525(6)	104.626(10)
*β*/°	88.785(9)	86.249(5)	73.587(5)	91.630(8)
*γ*/°	77.104(10)	80.269(6)	74.459(6)	90.774(8)
*V*/Å^3^	1124.9(2)	1170.3(1)	1260.1(1)	1316.6(2)
*Z*	2	2	2	2
*T*/K	293(2)	293(2)	293(2)	293(2)
*λ*_Mo_/Å	0.71073	0.71073	0.71073	0.71073
*ρ_cal_*_c_/g·cm^–3^	1.333	1.321	1.301	1.290
*F(000)*	476	492	524	544
µ/mm^−1^	0.097	0.095	0.093	0.094
*θ* range/°	3.34–25.01	3.54–25.01	3.44–25.01	3.19–25.01
Completness *θ*/%	99.7	99.7	99.8	99.7
Reflections collected	7273	7669	17038	8181
Reflectionsunique	3954[R_int_ = 0.0323]	4112[R_int_ = 0.0168]	4430[R_int_ = 0.0270]	4628[R_int_ = 0.0526]
Data/restraints/parameters	3954/0/340	4112/4/347	4430/2/375	4628/2/392
Goodness of fit on *F^2^*	1.020	1.014	1.042	0.996
Final R_1_ value (*I*>2σ(*I*))	0.0542	0.0458	0.0502	0.0667
Final *w*R_2_ value (*I*>2σ(*I*))	0.1020	0.1194	0.1242	0.1090
Final R_1_ value (all data)	0.1017	0.0593	0.0667	0.1909
Final *w*R_2_ value (all data)	0.1205	0.1298	0.1351	0.1544
CCDC number	1954713	1954715	1954714	1954716

**Table 2:** Hydrogen bonds geometry for compounds 1–4.

Compound	D–H···A	*d*(D–H) [Å]	*d*(H···A) [Å]	*d*(D⋯A) (Å)	∠D–H⋯A (°)
**1**	N(10)–H(10)···O(28)	0.91(2)	1.96(2)	2.865(3)	172(3)
	N(15)–H(15A)···O(27) ^i^	0.92(3)	2.11(3)	2.961(3)	155(2)
	N(15)–H(15B)···O(32)	0.93(3)	2.05(3)	2.951(9)	159(2)
	N(15)–H(15B)···O(32A)	0.93(3)	2.02(5)	2.91(5)	161(3)
	N(16)–H(16A)···O(27) ^ii^	0.96(3)	2.05(3)	2.979(3)	163(2)
	N(16)–H(16B)···O(31) ^iii^	0.87(3)	2.12(3)	2.975(3)	167(2)
	O(32)–H(32)···O(31) ^iv^	0.89(6)	1.90(6)	2.754(9)	160(6)
	C(1)–H(1)···O(32A)	0.93	2.56	3.46(4)	161
	O(28)–H(28)···O(30)	1.26(3)	1.15(3)	2.406(2)	176(3)
Symmetry code: (i) −x,1−y,1−z; (ii) x,−1+y,z; (iii) −x,1−y,−z; (iv) x,y,1+z.					
**2**	N(10)–H(10)···O(28)	0.88(2)	2.00(2)	2.871(2)	171(2)
	N(15)–H(15A)···O(27) ^i^	0.91(2)	2.10(2)	2.952(2)	154(2)
	N(15)–H(15B)···O(32)	0.90(2)	2.03(2)	2.906(4)	163(2)
	N(15)–H(15B)···O(32A)	0.90(2)	2.06(3)	2.931(18)	162(2)
	N(16)–H(16A)···O(31) ^ii^	0.89(2)	2.09(2)	2.961(2)	168(2)
	N(16)–H(16B)···O(27) ^iii^	0.86(3)	2.14(3)	2.974(2)	164(2)
	O(32)–H(32)···O(31) ^iv^	0.82	2.23	2.789(5)	126
	C(1)–H(1)···O(32)	0.93	2.58	3.455(4)	157
	C(1)–H(1)···O(32A)	0.93	2.57	3.433(2)	154
	O(28)–H(28)···O(30)	1.31(3)	1.08(3)	2.392(2)	173(3)
Symmetry code: (i) −x,1−y,1−z; (ii) −x,1−y,−z; (iii) x,−1+y,z; (iv) x,y,−1+z.					
**3**	N(10)–H(10)···O(32)	0.90(2)	1.96(2)	2.847(5)	169(2)
	N(10)–H(10)···O(32A)	0.90(2)	1.88(2)	2.772(1)	173(2)
	N(15)–H(15A)···O(27)	0.81(2)	2.13(2)	2.860(2)	150(2)
	N(15)–H(15B)···O(30) ^i^	0.90(3)	2.04(3)	2.870(3)	153(2)
	N(16)–H(16A)···O(31) ^ii^	0.87(2)	2.06(2)	2.918(3)	168(2)
	N(16)–H(16B)···O(31) ^iii^	0.89(3)	2.53(3)	3.108(3)	123(2)
	O(32)–H(32)···O(27) ^iv^	0.82	1.94	2.728(6)	162
	O(28)–H(28)···O(30)	1.21(3)	1.19(3)	2.388(2)	169(3)
Symmetry code: (i) 1−x,1−y,2−z; (ii) 1+x,y,−1+z; (iii) 2−x,−y,2-z; (iv) 2−x,1−y,1−z.					
**4**	N(10)–H(10)···O(37)	0.94(4)	1.87(4)	2.779(6)	164(3)
	N(15)–H(15A)···O(27)	0.87(4)	2.33(4)	3.171(5)	162(3)
	N(15)–H(15B)···O(32)	1.03(4)	1.97(4)	2.976(1)	165(3)
	N(15)–H(15B)···O(32A)	1.03(4)	1.91(5)	2.92(3)	167(3)
	N(16)–H(16A)···O(31) ^i^	0.93(5)	2.51(5)	3.348(6)	150(3)
	N(16)–H(16B)···O(31) ^ii^	0.96(5)	2.09(5)	3.037(6)	169(4)
	O(32)–H(32)···O(30) ^iii^	0.82	2.57	3.330(1)	154
	O(37)–H(37A)···O(27) ^iv^	0.72(7)	2.18(7)	2.878(5)	165(8)
	O(37)–H(37B)···O(28) ^i^	0.87(7)	1.97(7)	2.812(5)	163(6)
	C(8)–H(8)···O(27)	0.93	2.45	3.376(5)	174
	O(28)–H(28)···O(30)	1.26(6)	1.11(6)	2.371(6)	175(5)
Symmetry code: (i) x,−1+y,z; (ii) 2−x,2−y,2−z; (iii) 2−x,2−y,1−z; (iv) 2−x,1−y,1−z.					

**Table 3:** π–π interactions for compounds 1–4 (distance in Å and angles in degrees).

Compound	CgI ^a^	CgJ ^a^	CgI···CgJ ^b^	Dihedral Angle ^c^	Interplanar Distance ^d^	Offset ^e^
**1**	1	1 ^i^	3.806(1)	0.0(1)	3.402(1)	1.708
	1	2 ^v^	3.952(1)	1.5(1)	3.554(1)	1.732
	1	3 ^i^	3.689(2)	3.4(1)	3.458(1)	1.321
	2	2 ^v^	4.001(2)	0.0(1)	3.566(1)	1.814
	2	3 ^i^	3.913(1)	4.8(1)	3.570(1)	1.880
Symmetry code: (i) −x,1−y,1−z; (v) 1−x,1−y,1−z.						
	1	1 ^i^	3.709(1)	0.0(1)	3.370(1)	1.550
**2**	1	3 ^i^	3.615(1)	2.3(1)	3.404(1)	1.200
	3	2 ^i^	3.800(1)	3.1(1)	3.498(1)	1.483
Symmetry code: (i) −x,1−y,1−z.						
	1	1 ^iv^	3.871(1)	0.0(1)	3.386(1)	1.877
**3**	1	3 ^iv^	3.568(1)	1.7(1)	3.409(1)	1.027
	2	3 ^iv^	3.948(1)	3.4(1)	3.499(1)	2.020
Symmetry code: (iv) 2−x,1−y,1−z.						
	1	1 ^iv^	3.618(2)	0.0(2)	3.394(1)	1.252
**4**	1	3 ^iv^	3.743(2)	2.5(2)	3.377(1)	1.473
	2	3 ^iv^	3.731(2)	4.2(2)	3.495(1)	1.411
Symmetry code: (iv) 2−x,1−y,1−z.						

^a^ Cg represents the centre of gravity of the rings as follows (Figure 1): Cg1 ring C9/C11/C12/N10/C14/C13, Cg2 ring C1–C4/C12/C11, Cg3 ring C5–C8/C13/C14. ^b^ Cg···Cg is the distance between ring centroids. ^c^ The dihedral angle is that between the mean planes of CgI and CgJ. ^d^ The interplanar distance is the perpendicular distance from CgI to ring J. ^e^ The offset is the perpendicular distance from ring I to ring J.
